# A Retrospective Long-Term Follow-Up of the Randomized Study: Total Endoscopic Ablation of Patients With Long-Standing Persistent Atrial Fibrillation

**DOI:** 10.1093/icvts/ivag004

**Published:** 2026-01-08

**Authors:** Anders Wickbom, Espen Fengsrud, Anders Ahlsson

**Affiliations:** Department of Cardiothoracic and Vascular Surgery, Örebro University Hospital, Örebro, SE-70185, Sweden; Department of Medical Sciences, Faculty of Medicine and Health, Örebro University, Örebro, SE-70182, Sweden; Department of Medical Sciences, Faculty of Medicine and Health, Örebro University, Örebro, SE-70182, Sweden; Department of Cardiology, Örebro University Hospital, Örebro, SE-70185, Sweden; Department of Medical Sciences, Faculty of Medicine and Health, Örebro University, Örebro, SE-70182, Sweden

**Keywords:** long-standing persistent atrial fibrillation, sinus rhythm, thoracoscopic ablation, long-term follow-up

## Abstract

**Objectives:**

Minimally invasive surgical ablation for atrial fibrillation is an alternative to catheter ablation. Achieving a lasting sinus rhythm in long-standing persistent atrial fibrillation is challenging, and long-term data after surgery are limited. In 2016, we published a randomized trial comparing totally endoscopic box lesion ablation of the left atrium (case) to medical therapy (control) during 1 year in patients with long-standing persistent atrial fibrillation. This study presents data from a follow-up of our previous cohort to investigate the rhythm outcome long-term.

**Methods:**

This was an observational follow-up study. Most recent heart rhythm, time from totally endoscopic ablation to first relapse in atrial fibrillation, re-ablation, stroke, medication, left ventricular ejection fraction, and mortality were gathered from medical records and analysed with descriptive statistics and survival analysis.

**Results:**

At the end of the randomized trial, 80% of the cases had sinus rhythm without antiarrhythmic drugs. During a mean follow-up of 9 years, 43% of cases and 74% of controls had undergone additional ablation procedures (*P =* .009). At the last follow-up, 21% of cases and 5% of controls were in SR. After totally endoscopic ablation, the mean time from surgery to first relapse in atrial fibrillation was 23 months (14-31 [95% CI] *P* < .001).

**Conclusions:**

In this population of patients with long-standing persistent atrial fibrillation, totally endoscopic box lesion ablation of the left atrium had short-term efficacy in restoring sinus rhythm. Long-term efficacy could not be demonstrated, with a high proportion of relapse in atrial fibrillation beyond 1 year post ablation.

## Graphical abstract

**Figure:**
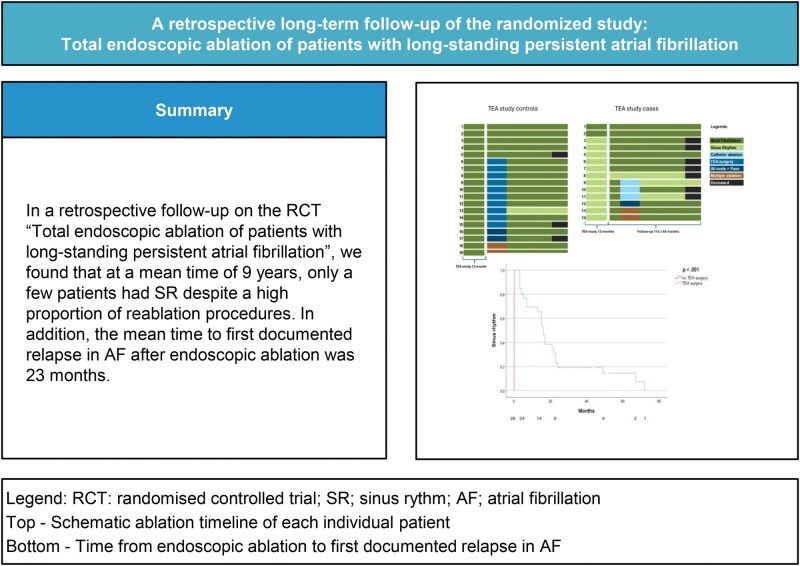

## INTRODUCTION

Atrial fibrillation (AF) is the most common cardiac arrhythmia worldwide and is associated with an increased risk of cardiac and cerebrovascular morbidity and mortality.^1^^,^^2^ Initial treatment after individual risk assessment entails mitigation of associated comorbidities, symptom management by rhythm or rate control, and risk reduction of thromboembolic events with anticoagulation.^3^

For symptomatic patients, catheter ablation can improve quality of life, reduce recurrence and progression,^4^^,^^5^ and is recommended at Class I LoE A for those in paroxysmal and persistent AF.^3^ However, the probability of successful ablation decreases with more advanced stages of AF. In persistent forms, long-term freedom from AF ranges from 20% to 50% and often requires multiple procedures.^6–8^

Thoracoscopic epicardial ablation is an alternative approach with favourable results in persistent AF compared to catheter ablation.^9^ Assessed in long-term follow-up, it is recommended with a class IIa LoE A indication in current guidelines.^3^ Achieving a lasting sinus rhythm (SR) is challenging in long-standing persistent AF (LSPAF); observational studies and randomized trials have reported heterogeneous success rates.^10–14^ In 2016, we published a small-scale Swedish single-centre RCT in patients with LSPAF, comparing a thoracoscopic epicardial ablation procedure with a left atrial box lesion versus non-ablation standard care. At 12 months, 80% of the operated patients remained in SR without antiarrhythmic medication (AAD), with superior quality of life (QoL) assessed by generic instruments (SF-36) and an increase in left ventricular ejection fraction (LVEF).^15^ This study presents data from an observational follow-up of the 2016 study cohort, to investigate the long-term heart rhythm outcome descriptively in each group.

## METHODS

### Patients, design, and end-points

The present study comprises a follow-up of patients who concluded their participation in the “Total endoscopic ablation of patients with long-standing persistent atrial fibrillation: a randomized controlled study” (TEA study).^15^ The TEA study had enrolled 36 patients aged >50 years with symptomatic LSPAF which were randomized to endoscopic epicardial ablation (case *n* = 17) or rate control therapy (control *n* = 19). Exclusion criteria in the randomized trial were left atrial thrombus formation, intolerance to oral anticoagulation (OAC), forced expiratory volume <1.5, left atrial anteroposterior diameter <60 mm, BMI <35, and/or previous cardiac surgery. All patients were monitored with a Reveal XT Insertable Cardiac Monitor (ICM) (Medtronic, Minneapolis, MN, United States). The surgical ablation procedure has previously been described.^16^ An epicardial left atrial box lesion was performed through a right-sided endoscopic approach, with temperature-controlled uni- and bipolar radiofrequency ablation using the Cobra Adhere XL catheter (Estech, Inc., San Ramon, CA, United States). Post-study, the patients’ individual physician continued follow-up and arrhythmia-specific healthcare, such as prescription of rate and rhythm control, OAC and, if indicated, referral for additional ablation. All patients from the control group were subsequently offered isolated TEA surgery after the conclusion of the TEA study with non-systematic postop antiarrhythmic treatment.

The present study comprises a retrospective observational follow-up of patients who concluded their participation in the TEA study (case *n* = 15 and controls *n* = 19). The primary end-point was time from TEA surgery to first relapse in AF. Secondary end-points were re-ablation procedures and all-cause mortality. A predefined case report form was used to gather variables of interest. Baseline data and current heart rhythm information were collected from digital hospital records: AF relapse, re-ablation (catheter ablation, surgical ablation, AV node ablation), rhythm and rate control, OAC, all-cause mortality, and left ventricular ejection fraction (LVEF).

### Ethical statement

The study was conducted in accordance with the principles of the Declaration of Helsinki.^17^ All data were pseudonymized and handled in compliance with the European Union General Data Protection Regulation (GDPR) and Swedish data protection laws. The study was approved by the Swedish national ethics board (EPM 2025-01871-02, EPM 2024–05712-01). In accordance, patients were informed of the study in writing and offered a choice of non-participation. This study does not comprise storage or biological material for multiple and indefinite use.

### Arrhythmia and re-ablation procedures

Arrhythmia was determined by 12-lead electrocardiogram (ECG) recordings; the most recent rhythm was analysed, as well as the date of first relapse in AF (if operated with TEA surgery). A 3-month blanking period after TEA surgery was applied. Catheter ablation was defined as any endocardial ablation (not only mapping) and endoscopic ablation as surgical ablation using the same technique as in the original randomized study^15^^,^^16^; AV node ablation was recorded separately from catheter ablation procedures. Multiple ablation procedures were recorded separately if procedures were consecutively with different ablation modalities.

### Statistical analysis

Categorical data are presented as frequencies and percentages, and continuous data are expressed as mean ± standard deviation (SD) or median (interquartile range). Categorical data were analysed using *χ*^2^ test or Fisher’s exact test, and Mann-Whitney *U*-test was used to analyse variables with non-normal distribution. Kaplan-Meier survival plots and log-rank test were used to investigate and illustrate time from surgical ablation to AF relapse for the original TEA case group (**Figure 2**) and for all patients that at any point underwent TEA surgery (original TEA case group + TEA controls that underwent TEA surgery after study conclusion) (**Figure 3**). All statistical analyses were performed using SPSS, version 22 (IBM Corp., Armonk, NY, United States).

## RESULTS

**Table 1** presents detailed patient characteristics at the time of enrolment in the TEA study. Most patients were men, with a median age of 66 years and a median AF duration of 4 years.

**Table 1.** Baseline Characteristics at Time of Enrolment in the TEA Study

	TEA case (*n* = 15)	TEA control (*n* = 19)
Age (years)	66.6 ± 5.5	65.8 ± 5.0
Gender (M/F)	13/2	13/6
BMI (kg/m^2^)	29.7 ± 3.5	27.5 ± 3.2
Hypertension	10 (67)	4 (21)
Diabetes mellitus	5 (33)	2 (10)
Chronic obstructive pulmonary disease	2 (13)	1 (5)
Obstructive sleep apnoea syndrome	1 (7)	1 (5)
TIA/stroke	1 (7)	2 (10)
Previous myocardial infarction	2 (13)	0
Congestive heart failure	7 (47)	7 (37)
Left atrial diameter (mm)	47 ± 5	43 ± 5
Left atrial area (cm^2^)	28.6 ± 5.7	26.7 ± 4.0
Left ventricular ejection fraction (%)	53 ± 9	56 ± 8
Left ventricular end-systolic diameter (mm)	38 ± 7	34 ± 6
Left ventricular end-diastolic diameter (mm)	52 ± 6	49 ± 6
Physical working capacity (% of expected)	94 ± 21	93 ± 13

Data are mean ± SD or *n* (%).

Abbreviations: BMI: body mass index; M/F: male/female; TIA: transient ischaemic attack.

**Figure 1.**
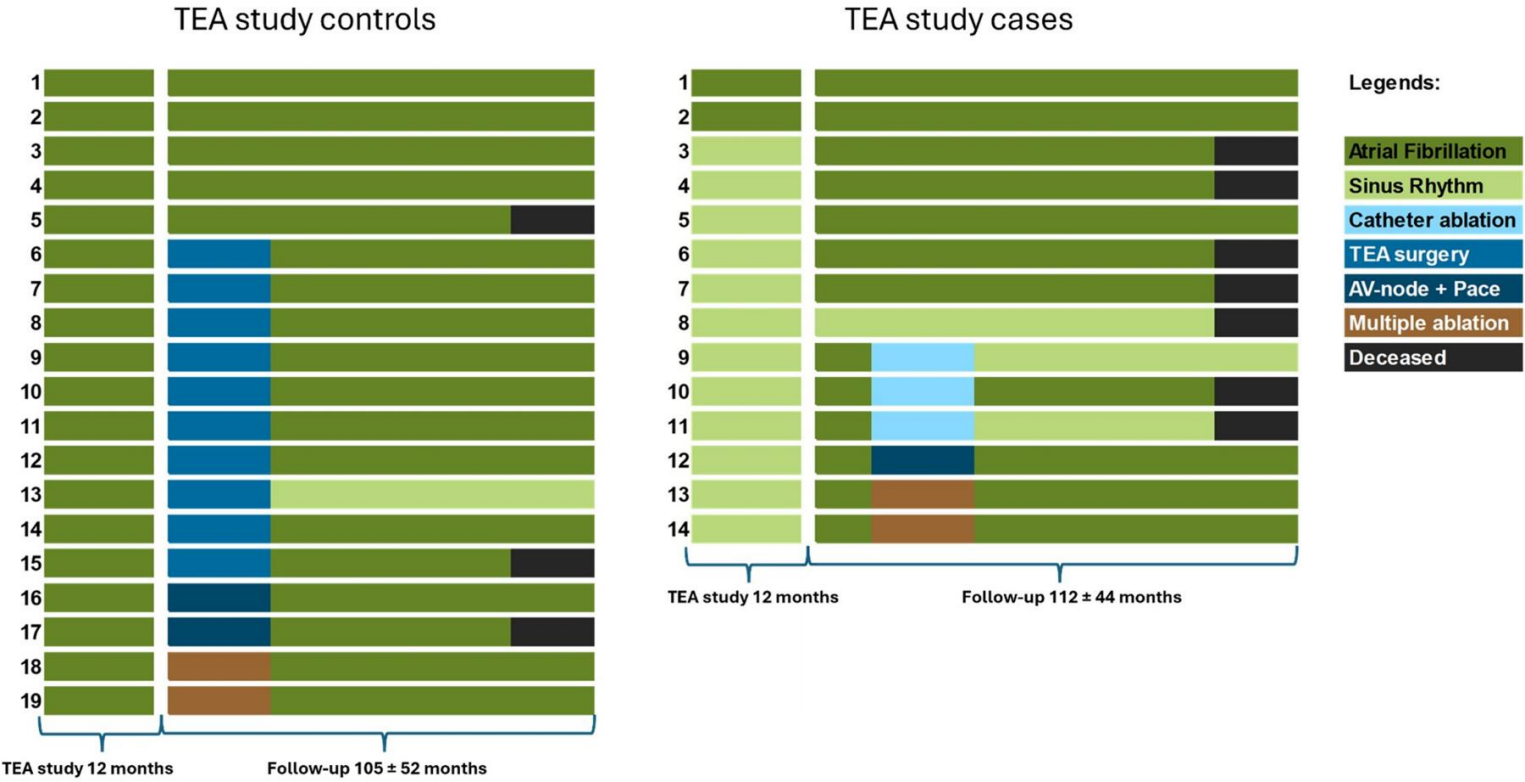
Rhythm Outcome Illustration.

One patient from the original TEA case group was lost to follow-up, leaving 14 TEA case group patients and 19 TEA control group patients. **Figure 1** shows an arrhythmia timeline illustration for each individual patient who was available to follow-up, according to their group status in the TEA study. Mean time of follow-up was 9 years; 112 ± 44 months for the TEA case group and 105 ± 52 months for the control group. At the most recent follow-up, 3/14 (21%) and 1/19 (5%) had SR (*P =* .193) (**Table 2**). There had been 7 deaths in the TEA case group (3 from malignancy, 1 from COVID-19, 1 due to non-ST elevation myocardial infarction, and 2 from unknown causes) and 3 deaths in the TEA control group (1 from stroke and 2 from unknown causes). After the TEA study ended, 6/14 (43%) in the TEA case group and 14/19 in the control group (74%) received additional ablation procedures (*P* = .009) (**Table 2**, **Figure 1**). Stroke occurred in 4 patients (2 in each group, respectively), of whom 1 patient from the TEA control group did not have ongoing OAC due to chronic subdural haematoma and 3 patients were using OAC at the time of stroke.

**Table 2.** Long-Term Follow-Up of the TEA Study Cohort

		TEA case (*n* = 14)	TEA control (*n* = 19)	*P*
Mean f-u time (months)		112 ± 44	105 ± 52	
All-cause mortality		7 (50)	3 (16)	.042
SR at most recent f-u		3 (21)	1 (5)	.193
Re-ablation after TEA study	None	8 (57)	5 (26)	
	Catheter	3 (21)	1 (5)	
	TEA surgery	0	8 (42)	
	AV node ablation	1 (7)	3 (16)	
	Multiple^a^	2 (14)	2 (11)	.009
Stroke after the TEA study end		2 (14)	2 (11)	.574
Antiarrhythmics		2 (14)	0	.172
Beta-block		14 (100)	15 (79)	.095
OAC/DOAC		14 (100)	17 (90)	.324
LVEF^b^	45-60%	11/12 (92)	16 (84)	
	30-44%	1/12 (8)	3 (16)	.632

Data are mean ± SD or *n* (%).

Abbreviations: f-u: follow-up; LVEF: left ventricular ejection fraction; OAC/DOAC: oral anticoagulation/direct oral anticoagulants; SR: sinus rhythm; TEA: totally endoscopic ablation.

a TEA case group: 2 patients—catheter ablation + AV node ablation. TEA control group: 1 patient—TEA surgery + catheter ablation + AV node ablation, 1 patient—TEA surgery + catheter ablation.

b In the TEA case group, 2 patients had not performed echocardiography after the TEA study ended.

**Figure 2.**
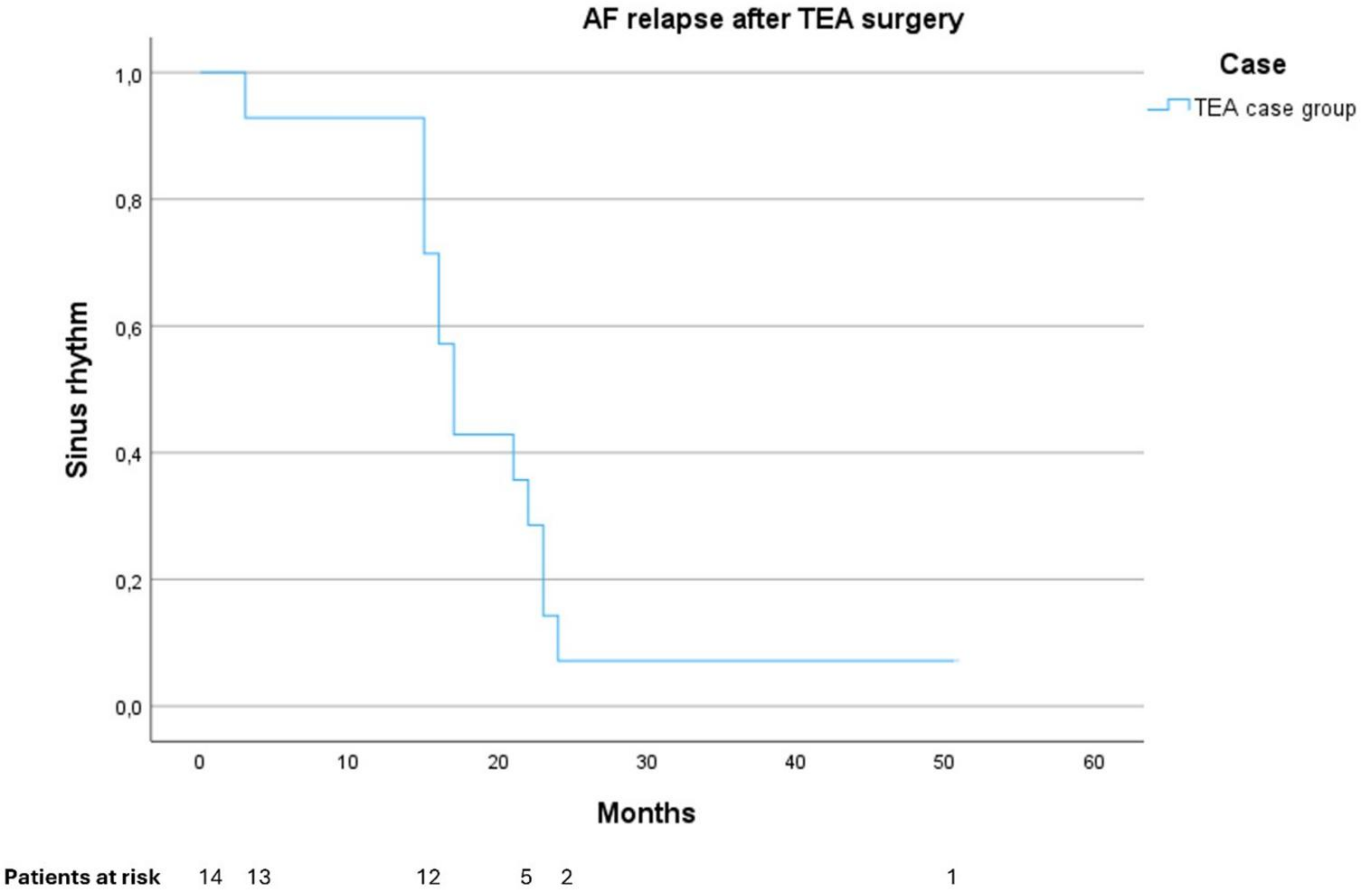
Time to First Atrial Fibrillation Relapse After TEA Surgery, TEA Study Case Group Only.

**Figure 2** shows the time from ablation/TEA study conclusion to first relapse in AF for the TEA case group only. Mean time for freedom from AF was 20 months (15-25 [95% CI], *P <* .001) (**Figure 2**).

**Figure 3.**
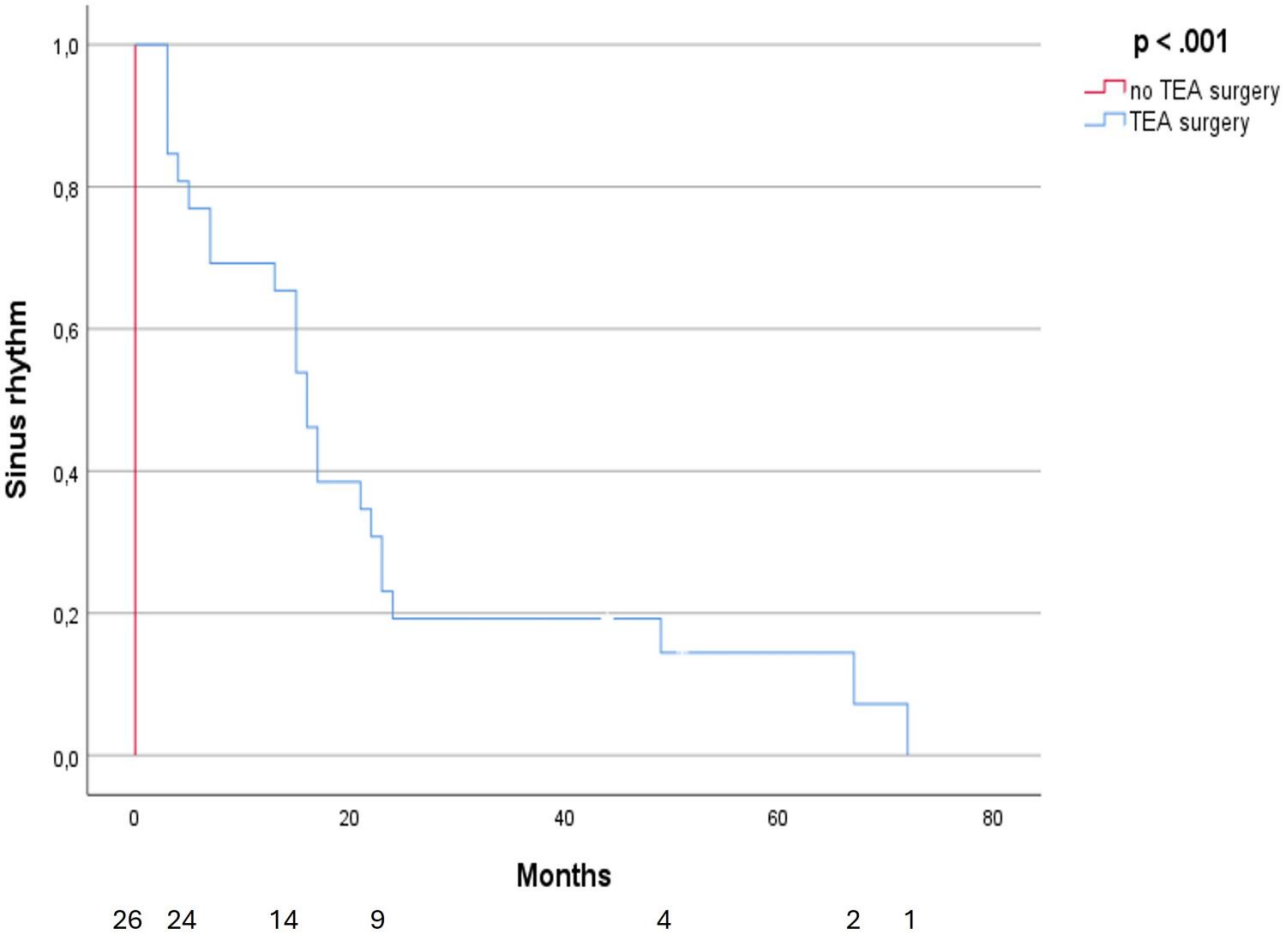
Time to First Atrial Fibrillation Relapse After TEA Surgery vs No TEA Surgery.

**Figure 3** shows the time from ablation to first relapse in AF for all patients that had undergone TEA surgery (TEA study cases + TEA study controls who received TEA surgery after TEA study conclusion). Mean time for freedom from AF was 23 months (14-31 [95% CI] *P* < .001).

## DISCUSSION

In this observational follow-up of the randomized TEA study cohort, our main finding was that a totally thoracoscopic box lesion of the left atrium by RF ablation was effective in short-term restoration of SR. Long-term benefits could not be demonstrated, with a mean time of 23 months in AF-free SR. Despite a high proportion of additional ablation procedures, AF recurrence was high in this population with LSPAF.

Previous data on the long-term efficacy of stand-alone surgical ablation in patients with LSPAF is limited to observational data and one randomized controlled trial.^11–13^ The studies show significant heterogeneity with differing surgical modalities, mixed populations with persistent AF and LSPAF, varying systematics in rhythm follow-up and to some extent non-systematic administration of postoperative AADs. As such, generalization and comparison between studies is challenging and risks overinterpretation. Ad’s group reported a 5-year follow-up of a version of Cox-maze IV ablation, on a mixed population of persistent and LSPAF, where freedom from AF without AADs was 73% and a cumulative freedom from AF recurrence was 58.5%.^11^ Lapenna *et al.* reported a 7-year follow-up, on a cohort of patients with persistent or LSPAF who underwent stand-alone Cox-maze IV surgery through either a midline sternotomy or a right-side mini-thoracotomy. The proportion of patients in SR without AADs was 74%; 26.5% had a cumulative incidence of AAD-free AF recurrence.^13^ Harlaar et al.^14^ reported freedom from atrial arrhythmia without AADs of 50% at a median of 5 years after bilateral thoracoscopic left atrial epicardial ablation for LSPAF, and a substantial de-escalation of AF severity. In contrast, the randomized controlled CASA-AF trial, comparing thoracoscopic left atrial epicardial ablation versus bi-atrial catheter ablation, showed that freedom of AF without AADs after surgical ablation was 26% after 12 months and 11% after 36 months.^10^^,^^12^ The surgical technique employed in our study resembles both CASA-AF and Harlaar et al., where the ablation was performed thoracoscopically epicardial, on a beating heart and confined to the left atrium, ableit with a less extensive lesion pattern. Although in our trial the proportion of patients in SR without AADs at 12 months was 80%, long-term persistence of SR for most of the patients could not be demonstrated. Mirroring the follow-up time in CASA-AF, freedom from AF recurrence 36 months after TEA surgery was <20%. The significant difference from a high proportion of patients free from AF long-term, demonstrated by Ad^11^ and Lapenna^13^ could be explained by the more extensive (Cox-maze IV) lesion sets in those studies. The Cox-maze IV procedure is a bi-atrial open-heart ablation with a standardized lesion set designed to address endo and epicardial AF triggers as well as wandering wavelets. This procedure may be needed if atrial remodelling in LSPAF has progressed to the extent that an isolated left atrial epicardial ablation strategy is insufficient for long-term restoration of SR. Hybrid ablation (HA) therapies combining epicardial with endocardial ablation in single or 2-stage sessions could theoretically bridge such a gap. A few randomized trials comparing HA to catheter alone have shown SR of 67%-89% after HA, without AADs.^18–20^ However, generalization on its efficacy is limited by short (12 month) follow-up and non-uniform AF types (mixed persistent and LSPAF) and lesion sets in the reported hybrid approaches. A recently published single-centre RCT comparing HA to thoracoscopic ablation in patients with non-paroxysmal AF showed superior results of HA with a 12-month freedom from AF without AADs of 73% compared to 43% after thoracoscopic ablation.^21^ Results are thus intriguing but still lacking long-term follow-up, which is warranted.

Risk reduction of thromboembolic stroke is a cornerstone in lifetime AF management. Closure of the left atrial appendage (LAA) is routinely performed as an adjunct in contemporary surgical ablation procedures.^22–24^ When performed concomitantly with cardiac surgery, LAA closure significantly reduces stroke risk long-term in a real-world population of patients with prior AF.^25^ One recent randomized trial has shown endovascular LAA closure during catheter ablation to be non-inferior to OAC treatment in terms of post-procedural long-term stroke risk.^26^ However, no studies have systematically investigated long-term reduction of stroke risk after surgical ablation procedures, and current guidelines do not support discontinuation of OAC after thoracoscopic ablation with LAA occlusion in patients with high or medium stroke risk determined by CHA2DS-VASc score.^3^ In the FAST trial comparing thoracoscopic ablation, including LAA exclusion versus catheter ablation without LAA occlusion, cerebrovascular events were similar (8% and 10%, respectively) during a mean 7-year follow-up period.^9^ In the long-term observational study by Lapenna *et al.*, where the LAA was routinely excluded surgically concomitant to ablation, stroke occurred in 0.8% of patients despite discontinuation of OAC in 81% of patients,^13^ but lack of standardization or randomization precludes conclusions beyond generation of hypotheses. A meta-analysis of RCT and observational data comparing thoracoscopic ablation with LAA exclusion vs catheter ablation with LAA occlusion found no significant short-term difference in stroke rates.^27^ LAA closure was not performed in the TEA study. Stroke occurred in 12% (*n* = 4) of the patients, including 3 with OAC therapy and 1 without OAC due to chronic subdural haematoma. As LAA exclusion could potentially be a significant benefit in thoracoscopic ablation procedures, its isolated role in long-term stroke risk reduction warrants further investigation.

Long-term freedom from AF in patients with LSPAF has been reported to increase with multiple catheter ablation procedures and can reach 80%.^6–8^ Re-ablation with minimally invasive surgical techniques is not recommended after surgical ablation, due to the development of postoperative adhesions; endocardial catheter ablation in Sweden is available only at selected highly specialized cardiac units. In Harlaar et al.’s study, postoperative endocardial touch-up procedures improved freedom from atrial arrhythmia.^14^ Judicious management of AF related risk factors plays an important part in overall AF management and is associated with improved ablation outcomes.^28^^,^^29^ In the original TEA case group, re-ablation was rarely utilized, and patients were equivalently subject to AV node ablation. Including TEA surgery as an additional ablation procedure post TEA study conclusion, the TEA control group had a high proportion of additional ablations, but multiple re-ablations were, however, less frequent in both groups. In all, it indicates that the underlying populations were at an advanced stage of LSPAF at the time of randomization. AF management after the TEA study was left to the discretion of the patient’s physicians and therefore non-systematic. In theory, substandard management of risk factors could explain the AF recurrence after the TEA study, but as detailed data on AF specific risk factor management were not available, this remains speculative.

Our study has several limitations. First, although the original study population was randomized, the present study design is retrospective, thus making the underlying data prone to bias. The post-TEA study non-systematic AF care increases the risk of confounding in the long-term rhythm, mortality, and other outcome results, which should be regarded as strictly observational. Second, heart rhythm follow-up was by 12-lead ECGs and thus cannot provide insights into AF burden and potential for AF recurrence. As increased arrhythmia burden and temporal AF pattern are associated with worse cardiovascular survival,^30^^,^^31^ reduction in AF burden and de-escalation of AF status are important outcome measures when assessing results after ablation therapies. Our data cannot provide such information, thus limiting its clinical relevance and further prohibiting any association to repeat ablation procedures, mortality, and morbidity outcomes. Last, the small patient population and the cross-over within the original TEA control group prohibit any comparison between the original groups, nor any viable possibility for regression modelling to account for confounding; results are therefore strictly observational and hypothesis-generating at best.

## CONCLUSION

In this population of patients with long-standing persistent atrial fibrillation, totally endoscopic box lesion ablation of the left atrium had short-term efficacy in restoring sinus rhythm. Long-term efficacy could not be demonstrated, with a high proportion of relapse in atrial fibrillation beyond 1 year post ablation.

## Data Availability

The data underlying this article will be shared on reasonable request to the corresponding author.
